# Establishing the Natural History and Growth Rate of Ameloblastoma with Implications for Management: Systematic Review and Meta-Analysis

**DOI:** 10.1371/journal.pone.0117241

**Published:** 2015-02-23

**Authors:** Michael P. Chae, Nicolas R. Smoll, David J. Hunter-Smith, Warren Matthew Rozen

**Affiliations:** 1 Department of Plastic and Reconstructive Surgery, Frankston Hospital, Peninsula Health, Frankston, Victoria, Australia; 2 Department of Surgery, Monash University, Clayton, Victoria, Australia; 3 Monash University Plastic and Reconstructive Surgery Unit, Peninsula Clinical School, Frankston, Victoria, Australia; 4 Department of Surgery, James Cook University Clinical School, Townsville, Queensland, Australia; Georgia Regents University, College of Dental Medicine, UNITED STATES

## Abstract

**Background:**

Ameloblastoma is the second most common odontogenic tumor, known to be slow-growing, persistent, and locally aggressive. Recent data suggests that ameloblastoma is best treated with wide resection and adequate margins. Following primary excision, bony reconstruction is often necessary for a functional and aesthetically satisfactory outcome, making early diagnosis paramount. Despite earlier diagnosis potentially limiting the extent of resection and reconstruction, an understanding of the growth rate and natural history of ameloblastoma has been notably lacking from the literature.

**Method:**

A systematic review of the literature was conducted by reviewing relevant articles from PubMed and Web of Science databases. Each article’s level of evidence was formally appraised according to the Centre of Evidence Based Medicine (CEBM), with data from each utilized in a meta-analysis of growth rates for ameloblastoma.

**Results:**

Literature regarding the natural history of ameloblastoma is limited since the tumor is immediately acted upon at its initial detection, unless the patient voluntarily refuses a surgical intervention. From the limited data, it is derived that the highest estimated growth rate is associated with solid, multicystic type and the lowest rate with peripheral ameloblastomas. After meta-analysis, the calculated mean specific grow rate is 87.84% per year.

**Conclusion:**

The growth rate of ameloblastoma has been demonstrated, offering prognostic and management information, particularly in cases where a delay in management is envisaged.

## Introduction

Ameloblastoma is the second most common, benign, but locally aggressive odontogenic tumor [1–3]. Most tumors arise from the mandible or maxilla, and affect between the third and fourth decade of life. Ameloblastoma can be clinically classified into solid, multicystic or unicystic or peripheral subtypes. The solid, multicystic type is the most common, while the unicystic type accounts for 5–15% of the cases, affects a younger population and has 3 variants: simple, luminal and mural. Peripheral ameloblastoma is the least common and has a benign biologic behavior. Ameloblastoma most often presents as a hard painless intraoral swelling or as an incidental finding on routine dental imaging. Although histologically benign, 2–4.5% of all cases have malignant potential and metastasize, most commonly to the lung [4,5]. Hence, the goal of management entails a complete excision with linear margins and early bony reconstruction. Adequate margins can be confirmed histologically postoperatively or radiologically with intraoperative imaging [1,6,7]. Long-term follow-up is critical since recurrences can occur up to 45 years after the initial resection.

Historically, ameloblastoma has been treated with curettage by community dentists or resected by surgeons before a detailed histological work-up is undertaken. In these settings, under-treatment and the persistent biologic behavior of ameloblastoma has resulted in high recurrence rates and morbidity. Given the potential for significant destruction of local anatomy, locoregional recurrences and metastatic potential, a clear understanding of the natural history of ameloblastoma is warranted. Such information can give a guide as to the urgency of management, guide treatment approaches and offer prognostic information. However, this understanding is notably absent from the literature.

In the current study, we have conducted a systematic review of the literature and a meta-analysis of 16 reports, from which the documented tumor dimensions and the duration of symptoms have been used to derive at a quantitative growth rate of ameloblastoma. Such understanding of the growth and natural history of ameloblastoma will be useful when offering treatment options at varying growth phases.

## Methods

### Search Strategy and Selection Criteria

**Fig 1:**
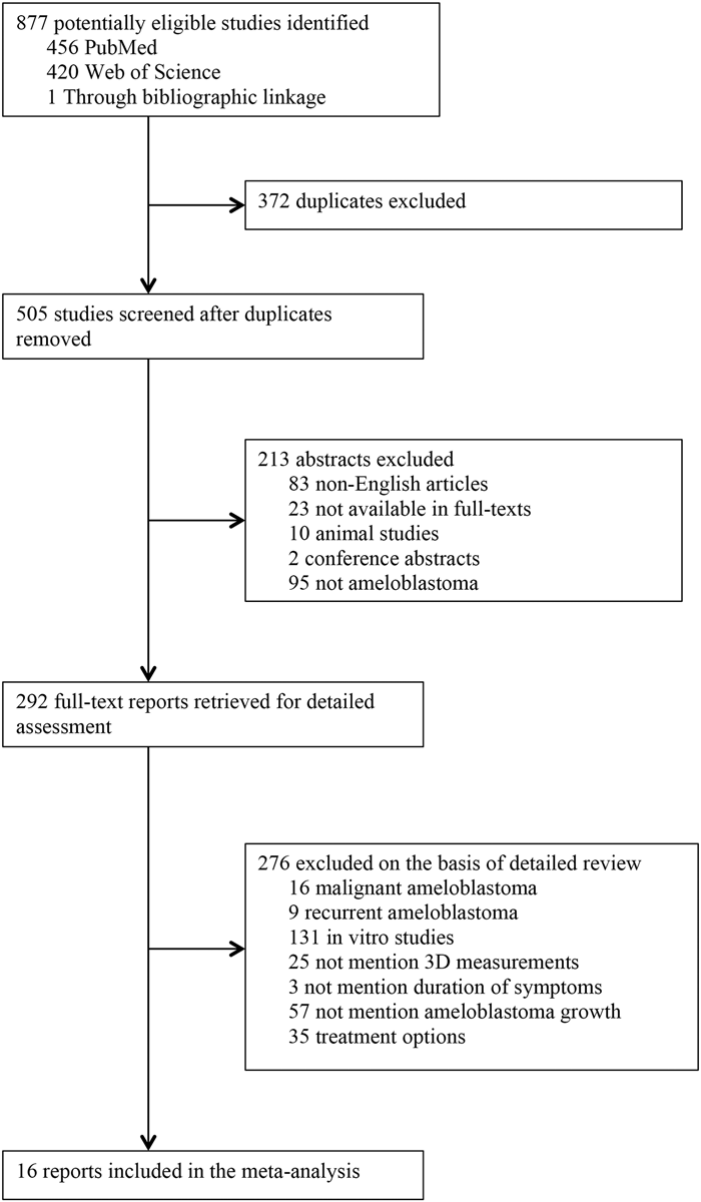
PRISMA (Preferred Reporting Items for Systematic Reviews and Meta-Analyses) flowchart for literature attrition in systematic review [8].

The current study comprises of a systematic review of the literature and a meta-analysis, aiming to establish the growth and natural history of ameloblastoma. We performed a comprehensive search of the databases including PubMed and Web of Science for eligible studies published between Jan 1, 1950, and May 27, 2014. Search terms were a combination of “ameloblastoma” with “growth”, “growth rate”, “natural history”, “untreated”, “declined surgery”, “giant”, or “extreme”. Additional references identified through the reference lists of selected references and bibliographic linkage were included in the review. A PRISMA (Preferred Reporting Items for Systematic Reviews and Meta-Analyses) flowchart for literature attrition is included (Fig. 1) [8]. Only papers published or translated in English were reviewed.

The following inclusion criteria were applied for the meta-analysis: human case reports/series of benign ameloblastoma before the initial surgical intervention and where all three dimensions of the tumor (i.e. length *x* width *x* height) and the duration of symptoms were reported. We excluded studies reporting the growth of ameloblastic carcinoma, malignant ameloblastoma, and recurrent ameloblastoma, in vitro cellular growth or molecular studies, reports where only one or two dimensions of the tumor volume were reported.

### Data Extraction

We developed a data abstraction sheet, to record necessary information to establish the level of evidence, study quality, and available outcome and risk factor details. Bias risk was evaluated and the level of evidence was assessed formally according to CEBM (Centre for evidence Based Medicine) evidence level. The CEBM (http://www.cebm.net) attributes standardized levels of evidence, from level 1a (systematic review of randomized control trials) to level 5 (expert opinion), to any research paper. Each of the included studies was thus critically appraised based on their study design and content. For meta-analysis, we recorded patient age, sex, tumor volume, mode of volume measurement, duration of symptoms, and histological subtype.

### Data Synthesis and Analysis

Two authors (MPC and WMR) independently screened records and assessed each retrieved full-text articles for inclusion. Disagreements were resolved by a blinded third reviewer (DJHS).

Studies inconsistently documented the tumor volume from either the surgical specimen, the plain radiograph, or from clinical examination. We considered the direct measurement from a surgical specimen the most accurate, then radiographs, and clinical examination in descending order. Hence, where the tumor volume was mentioned multiple times in a report, we would select the most accurate volume.

We extracted tumor volume dimensions from each study and the duration of symptoms in order to derive the specific growth rate (SGR; growth % per year) of each case. SGR was calculated using a formula previously described to quantify the tumor response to anti-cancer treatment [9]. Mehrara *et al* [9] mentions a logarithm of the ratio of post-treatment tumor volume (V_2_) to the pre-treatment tumor volume (V_1_) divided by the duration of treatment (T_2_-T_1_) (Equation 1).

$$SGR=\frac{ln(V_{2}/V_{1})}{T_{2}-T_{1}}$$ 1

For appropriate calculation in our study, V_1_ was considered “1” and T_1_ as “0”.

### Statistical Analysis

Statistical software, STATA (Version 13; StataCorp; College Station, TX, USA) was used for analysis. Initially, we treated SGR as a continuous variable and utilized Kruskal-Wallis equality-of-populations rank test. We calculated the statistical association between SGR and gender or three age groups (0–20, 21–40, 41 years and older). Then, we divided SGR into two groups: less than 100% per year and equal to or more than 100%. Using Pearson’s chi-squared test, we compared SGR against different histological subtypes (plexiform and follicular versus the rest), the three age groups, and gender. In all analyses, a p value lower than 0.05 was used as the level of statistical significance.

### Role of the Funding Source

There was no funding source for this study. All authors had full access to the data included in the study and had final responsibility for the decision to submit for publication.

## Results

### Literature Review

Systematic review of the literature identified 2 formal papers that discuss the natural history and growth rate of ameloblastoma. In a retrospective review of 100 cases of ameloblastoma, Odukoya *et al* report a superior average growth rate in the solid, multicystic subtype, compared to the peripheral subtype (0.81 vs 0.17 cm^3^/month respectively) [10]. In a similar study, the same authors have retrospectively analyzed the maximal tumor diameter from 330 biopsy specimens and found that the solid, multicystic type grows more aggressively than desmoplastic ameloblastomas (0.71 vs 0.36 cm/month respectively) [11].

Through rigorous assessment (Fig. 1), we also identified 16 published reports where the tumor volume and the duration of symptoms before receiving any treatment were known. They were utilized for the subsequent meta-analysis and to derive the SGR [12–27].

### Natural History and Growth Rate of Ameloblastoma

Throughout the literature, cases have been reported where patients with ameloblastoma did not receive immediate primary surgical interventions for various reasons—economic, fear of surgery, ignorance—and presented with a large solid, multicystic ameloblastoma [12–27]. Using the inclusion and exclusion criteria mentioned previously, we have identified and analyzed 16 published cases to calculate SGR of benign ameloblastoma (Table 1). The average age is 41 year-old (range: 10–62) and females are more frequently reported (2.67:1). There are six plexiform, two follicular, and one acanthomatous histological subtypes, and two are unknown. Mean duration of symptoms is 9.04 years (range: 0.17–23).

**Table 1:** Characteristics of large ameloblastoma cases reported in the literature.

	Age (year)	Sex	Volume (cm)	Weight (gram)	Mode of measurement	Duration of Symptoms (years)	SGR (%/year)	Histological Type	Level of Evidence and Study Quality
Hunasgi[23]	39	F	12.0 *x* 10.0 *x* 9.0	1200	Surgical	10	69.85	Granular cell	4
Catherine[24]	48	F	30.0 *x* 18.0 *x* 10.0	N/A	Radiographic	23	37.37	Follicular & plexiform	4
Mijiti[25]	40	M	25.0 *x* 20.0 *x* 15.0	N/A	Radiographic	15	59.48	Desmoplastic	4
Ota[22]	32	F	27.2 *x* 20.3 *x* 15.1	1600	Surgical	10	90.28	Acanthomatous	4
Chauhan[21]	42	F	15.0 *x* 14.0 *x* 10.0	N/A	Surgical	4.5	169.98	Plexiform	4
Acharya[26]	35	F	15.0 *x* 12.0 *x* 10.0	1350	Surgical	10	74.95	Plexiform	4
Hata[20]	53	M	14.0 *x* 11.0 *x* 10.0	N/A	Clinical	11	66.72	Follicular	4
Mukhopadhyay[27]	32	M	25.0 *x* 15.0 *x* 10.0	N/A	Surgical	7	117.56	N/A	4
Hughes[19]	53	F	15.2 *x* 11.4 *x* 12.0	1280	Surgical	6	127.32	Plexiform	4
Dunn[18]	62	F	17.0 *x* 15.0 *x* 13.0	1282	Radiographic	6	135.10	Plexiform	4
Gordy[17]	19	F	8.0 *x* 6.0 *x* 6.0	N/A	Surgical	5	113.19	Follicular	4
Ueyama[16]	73	M	10.0 *x* 9.0 *x* 7.5	435	Surgical	10	65.13	Plexiform	4
Nakasato[15]	39	F	11.0 *x* 10.0 *x* 6.0	386	Clinical	6	108.18	Plexiform	4
Pramulio[14]	10	M	9.0 *x* 6.0 *x* 5.0	N/A	Clinical	0.17	3356.83	Unknown	4
Osaki[13]	30	F	14.0 *x* 13.0 *x* 12.0	936	Surgical	7	109.84	Plexiform	4
Rambo[12]	41	F	21.0 *x* 15.0 *x* 15.0	N/A	Surgical	14	60.43	Unknown	4

Abbreviations: N/A: not applicable; SGR: specific growth rate.

We have only perused reports where all three dimensions of the tumor volume are known in order to attain the most accurate estimate of the volume for calculating SGR. We excluded studies of ameloblastoma carcinoma or malignant ameloblastoma since they display significantly different biological behaviors [4]. Ameloblastic carcinoma histologically exhibits malignant features. Even though histologically appear benign, malignant ameloblastoma tend to metastasize to distant sites in contrast to benign ameloblastomas. Furthermore, we have limited our search to the clinical cases and omitted studies of in vitro growth rate or expression of proliferative cell markers. They do not adequately account for the complex multi-factorial in vivo environmental elements contributing to the tumor growth.

Using the formula devised by Mehrara *et al* [28] for our meta-analysis, the mean SGR was initially calculated as 298% per year (range: 37.37–3356.83). Interestingly, once the outlier (3356.83% [14]) is removed, the mean SGR decreases significantly to a more reasonable 87.84% (range: 37.37–169.98). Pramulio *et al* [14] acknowledge that the patient’s history may have been unreliable and the duration of symptom may have been much longer.

### Factors Associated with More Rapid Growth

The calculated growth rates as above are based on averages from the reported series. This is clearly not a reflection of the tumor biology nor the intrinsic growth of ameloblastoma and its subtypes, but of the average calculated rates. We have analyzed our findings to identify factors that would be potentially associated with a higher SGR. Interestingly, none of the factors—gender, age groups, and histological subtypes—have shown statistical significance with SGR (Table 2). The most statistical significance is found where histological subtypes divided into two subgroups—plexiform and follicular versus the rest—are correlated to SGR also divided into two subgroups—less than 100% versus equal to or more than 100% (p = 0.14). This discrepancy is most likely due to the small sample size (n = 16). Unfortunately given the fact that most cases of ameloblastoma are almost universally treated immediately upon detection, it may be difficult to perform a study like this in a large scale.

**Table 2:** Summary of statistical analysis of patient factors against the specific growth rate of the tumor.

P values	Gender	Age group	Plexiform and follicular vs other histological subtypes
SGR as a continuous variable	0.24	0.68	N/A
SGR in 2 groups	0.31	0.25	0.14

Abbreviations: SGR: specific growth rate; vs: versus; N/A: not applicable.

Notwithstanding, in the literature, there are other factors that have established a clear correlation with more rapid growth and a poorer outcome. These include maxillary ameloblastoma when compared to the mandible [29], the solid, multicystic histological subtype, unicystic subtype invading the fibrous wall [30], older age (as young age is associated with unicystic ameloblastoma, and hence, better outcome) [31], malignant ameloblastoma, and suboptimal treatment (e.g. curettage, enucleation).

## Discussion

### Background

An understanding of the growth of ameloblastoma mandates a “control group” of ameloblastoma from which its natural history can be derived. For decades, ameloblastoma has been treated primarily surgically, either conservatively or radically, at the time of its first presentation without confirmatory histological diagnosis. This has meant that few studies offer any information about the untreated growth of these tumors. Furthermore, due to its rare nature, most reports have been limited to small-scale case studies. By combining data from all eligible published reports, we have derived at a quantitative growth rate of benign ameloblastoma. Despite the relatively small sample size (n = 16), the information will be useful for planning management, especially in the early stages of tumor growth.

While most authors would simply describe ameloblastoma as a slow growing tumor, a group in Nigeria has quantified its growth rate [10,11]. Odukoya *et al* [10] report a level 4 retrospective analysis of 100 ameloblastoma cases treated in a single center. They have calculated estimated monthly growth rate of the tumors in order to predict the “biologic aggression” of individual subtypes by dividing the average tumor volume at presentation to the duration of symptoms. Consistent with the literature, the solid, multicystic ameloblastoma has the fastest growth rate and the peripheral subtype the slowest (0.81 versus 0.17 cm^3^/month, respectively). Limitations of this study include the assumption that ameloblastoma follows linear growth. Furthermore, the mechanism of tumor volume measurement (e.g. clinically versus radiologically) and the reasoning behind the selection of 100 cases are not clarified. Moreover, the authors acknowledge their reliance on the patients to provide accurate history. Interestingly, the same authors [11] would later use the largest tumor diameter to compare the estimated growth rate of desmoplastic ameloblastoma and the conventional solid, multicystic type in another level 4 retrospective study of 330 biopsy specimens. The limitations are similar to the previous study but, in addition, the latter analyses only a small sample size of desmoplastic specimens (n = 14). They conclude that desmoplastic ameloblastoma may be less biologically aggressive compared to the solid, multicystic type (0.36 versus 0.71 cm/month, respectively) due to the desmoplasia acting as a tumor-limiting barrier.

For meta-analysis, we have used the formula mentioned by Mehrara *et al* [9]. In contrast to the more traditional tumor doubling time, SGR (Equation 1) more accurately reflects the natural exponential growth of the tumour [9]. Odukoya *et al* [10] and Effiom *et al* [11] simply divide the tumor volume or diameter by the symptom duration to arrive at an estimated monthly growth rate, which falsely assumes a linear growth pattern. In fact, evidences show that ameloblastoma initially exhibits slow growth, but later its growth accelerates [13,14,32]. Pramulio *et al* [14] reports a 10 year-old boy with a 2 month history of right jaw swelling that grows substantially in the few weeks prior to the presentation. Osaki *et al* [13] mentions a 30 year-old female with a 7 year history of left mandibular swelling that accelerates to the lower jaw 3 years before presentation. Rajaonarison Ny Ony *et al* [32] describes a case of maxillary ameloblastoma in a 23 year-old female growing slowly for 15 years before exhibiting accelerated growth in the 5 weeks preceding to the presentation.

Symptomatically, patients with ameloblastoma initially present with painless intraoral swelling. Early complications include generalized edema and anemia secondary to selective hypoproteinemia from transudation through the semipermeable cyst wall and bleeding from the ulcerations respectively. These are resistant to aggressive medical treatment. Later complications are associated with obstruction leading to small mouth opening, such as difficulty with mastication, deglutition, phonation and airway obstruction, as well as the loss of dentition on the ipsilateral side. It is worth noting that all of the metabolic derangements improved dramatically after radical tumor resection.

### Management of Benign Ameloblastoma

Despite its benign histology, ameloblastoma is associated with significant morbidities and is fatal if suboptimally treated [33,34]. Complete surgical removal of tumor and restoration of function and appearance are the main goal of therapy [6,35]. Surgical intervention is popularly classified into conservative resection with or without adjunctive therapy, and radical resection. Tumor excision is ideally followed by reconstruction with a bone graft or flap, distraction osteogenesis and dental prostheses [36]. Reconstruction is easier if done earlier due to absence of scarring or contracture, and it can be beneficial psychologically. Radiotherapy and chemotherapy have no role in ameloblastoma management. Radiation used alone is associated with 100% failure rate and serious complications, such as osteomyelitis leading to death and sarcoma development [37].

### Conservative Resection

Conservative surgical approach, such as curettage and enucleation, is frequently used for the treatment of unicystic ameloblastoma, except in the mural variant where the epithelium invades the cyst wall [3,38,39]. Although technically straightforward, curettage or enucleation can be associated with high recurrence rates (30–90%) when used against more aggressive solid, multicystic ameloblastomas [2,40–43]. Moreover, curettage or enucleation may not be able to remove the tumor tissue from within the cancellous bone beyond the macroscopic appearance and radiographic boundary. Hence, conservative resection has widely only been advocated in solid, multicystic ameloblastomas for patients of less than 10 years of age or smaller tumors [44]. Interestingly, evidence has also been offered that even for unicystic ameloblastomas, curettage or enucleation may still be associated recurrence rates (35–60%) higher than radical excision [35,45–48]. Furthermore, the luminal and intraluminal variants of unicystic ameloblastoma are difficult to differentiate from the mural type preoperatively, leading to the risk of “under-treatment” [49].

In order to improve its efficacy, curettage or enucleation can be paired with cryotherapy [50], electrocautery [51] or application of cauterizing agents like Carnoy’s solution [52]. Despite encouraging early results [53–55], the combination of curettage and cryotherapy has a recurrence rate (31%) higher than radical resection and is also associated with pathological fracture (11%) and wound dehiscence (30%) [50]. Hence, this is not appropriate where soft tissue extension and cortical thinning or perforations are present. The results are similarly unremarkable with electrocautery and Carnoy’s solution.

### Radical Resection

A radical approach—either marginal or segmental resection with adequate margin—ensures maximal removal of solid, multicystic ameloblastoma, minimizing the recurrence rate (0–10%) and the risk of metastasis [41,56–60]. Some literature suggests that ameloblastoma can extend into cancellous bone histologically at a mean of 4.5 mm (range: 2.3–8 mm) beyond the radiographic boundary [61] and currently, the literature recommends the use of 1–1.5 cm resection margin [48]. Satisfactory margins can be achieved by the application of new specialized imaging techniques, such as flat panel volumetric CT (fpvCT) [1], which can provide accurate anatomical details for intraoperative margin assessment.

In maxillary ameloblastoma, early radical resection is especially beneficial. Although less common and histologically similar to mandibular ameloblastoma, maxillary ameloblastoma acts clinically more aggressive. Maxilla lacks the thick cortical bone found in mandible that can slow down the tumor growth. Furthermore, maxillary ameloblastoma can potentially invade the central nervous system [29,40,62] and the rate of surgical cure decreases significantly once the tumor has extended beyond the confines of the maxillary bone [63]. In contrast, peripheral ameloblastomas are relatively innocuous with no bone involvement and can be sufficiently managed with a local excision and a long-term follow-up of the surgical site [64].

Marginal resection for solid, multicystic ameloblastoma is advantageous since it preserves the inferior margin of the mandible and prevents the necessity of a complex bone reconstruction [65]. However, where radical marginal resection may result in jaw instability and an increased risk of pathological fractures, segmental resection with bony reconstruction is the preferred option [6]. Furthermore, the unexcised inferior margin of mandible may present a source of tumor recurrence [65]. Jaw reconstruction techniques with a bone graft or a flap are now well described in the literature and are associated with relatively low morbidity [66].

### Bony Reconstruction

An understanding of the natural history of ameloblastoma can potentially limit the extent of resection and reconstruction. Conservative resection through enucleation or marginal resection can facilitate a limited reconstruction, with either no reconstruction at all, or bone graft reconstruction. In this setting, either autologous or alloplastic options are available, which can each fill the resection defect and provide form and/or strength to the remaining facial skeleton.

Where a segmental alveolar defect exists, in either mandible or maxilla, a bone graft can be used for smaller defects, particularly those less than 5 cm in length. A bone graft in this setting can be derived from the iliac crest (most commonly) [67,68], fibula [69], scapula [70], rib [71] or the radius [72]. A bone graft can be broadly classified as non-vascularized or vascularized. A non-vascularized bone graft heals by ‘creeping substitution’, in which there is a combination of osteoblast migration and bony scaffold graft take, while the framework maintains structural integrity during this process. Compared to alloplastic or vascularized options, bone grafts can provide a better bulk of bone for the placement of dental implants, a superior contour, undergoes remodeling upon placement and is associated with shorter hospital stays and decreased number of subsequent operations. Hence, a non-vascularized bone graft is indicated where the bony defect is shorter and an adequate amount of soft tissue is available. Handschel *et al* [73] recommends non-vascularized iliac crest graft for mandibular defects up to 5–6 cm in length. Since the average length of defect experienced by the author was 4.9 cm, the graft is suitable for most cases. Increased graft length is associated with an increased graft failure rate [74]. Pogrel *et al* [75] reports 75% failure rate for grafts longer than 12 cm and recommends extreme caution when using grafts longer than 9 cm. Additional advantages of a bone graft include being able to prepare and insert one in the same operation with the primary resection [76]. In addition, it provides a superior function in regard to mastication and deglutition [77]. Successful graft uptake is assessed by the maintenance of bone continuity, complete consolidation with the absence of infection examined clinically in the operating theatre or on imaging [78]. Potential complications are infection, fracture and plate exposure [73]. In addition, a variable degree of bony resorption is associated with bone grafts [56].

For a larger bony defect or where inadequate soft tissue is available, a free flap (vascularized bone graft) is suggested instead. The ability to vascularize a bone based on its intrinsic vasculature has meant that large segments, large enough to reconstruct the entire alveolus, can be transferred safely in a single stage. As for bone grafts, the iliac crest, fibula, scapula, rib and radius have all been described in this setting. These flaps can be transferred as pedicled or free flaps, and can also be transferred as bone only, or composite flaps. While useful in complex situations, these flaps mandate lengthy operative times, increased length of hospital stays and patient morbidity.

### Length of Follow-Up

Given the biologic behavior of ameloblastomas, a long-term follow-up is mandatory. More than 50% of recurrences occur within 5 years of the primary surgical intervention [3]. However, sporadic reports of recurrences at 20, 30 and 45 years have been reported in the literature [79–82]. Of note, higher recurrence rate is reported in granular and follicular histological subtypes [3]. Inadequately short follow-up may give physicians a false indication of cure and a potential to miss metastatic ameloblastoma [3].

## Conclusion

Current meta-analysis has produced a mean SGR of 87.84% growth per year for benign ameloblastoma, after removing outliers, which offers prognostic and management information, particularly in cases where a delay in management is envisaged. The greatest growth rate may be associated with plexiform and follicular histological subtypes, but this did not reach statistical significance. Early intervention can limit subsequent growth and facilitate more conservative reconstructive options.

## Supporting Information

S1 PRISMA Checklist (PDF)
